# An Unusual Presentation of Vivid Hallucinations

**DOI:** 10.7759/cureus.25441

**Published:** 2022-05-29

**Authors:** Arielle Degueure, Andee Fontenot, Ammar Husan, Muhammad W Khan

**Affiliations:** 1 Medicine, Louisiana State University Health Sciences Center, Shreveport, USA; 2 Neurology, University of Rochester Medical Center, Rochester, USA

**Keywords:** peduncular hallucinosis, charles bonnet syndrome, epilepsy, visual cortex, occipital, seizures, hallucinations

## Abstract

Visual hallucinations may present secondary to neurologic, psychologic, or physiologic disturbances. Certain features and characteristics of visual hallucination are often attributed to various brain regions; however, with a broad list of causes and multifaceted pathophysiology, it is often hard to accurately localize. Overlapping clinical presentations may be due to the pathology of brain interconnections, rather than isolated brain regions themselves. In this study, we discuss a case of isolated, complex visual hallucinations secondary to occipital seizures in the radiologic absence of an ischemic injury. We propose that a network-based localizing lesion is responsible for this unconventional presentation.

## Introduction

Visual hallucinations are typically a result of neuro-ophthalmologic dysfunction [1]. Some pathologies associated with this dysfunction include ocular disease, neurodegenerative disease, migraine aura, substance abuse or withdrawal, narcolepsy, brain tumors or abnormalities, and seizures [2].

Occipital cortex seizures are uncommon with estimates of only 2%-8% of patients experiencing this phenomenon [3]. Within the adult population, common underlying culprits include tumors, development abnormalities, and vascular malformations [4]. Although not always present, visual hallucinations are a hallmark feature of occipital seizures and are typically a seizure aura. Additionally, sensations of ocular movement, tinnitus, and vertigo can be manifestations and are thought to occur with spread to the posterior temporoparietal region. Other somatosensory and experiential phenomena may be experienced as well [2].

Visual hallucinations are broadly classified into two categories: simple (elementary or non-formed) hallucinations or complex (formed) hallucinations. Simple hallucinations manifest as lights, colors, lines, and shapes or geometric designs. Complex hallucinations differ in that these present as formed images of objects, animals, people, and lifelike scenes [5]. Traditionally, hallucinations initiated in the occipital lobe are described as elementary in nature, and these simple hallucinations are experienced as bright spots or simple geometric shapes, commonly restricted to one visual field [6]. Complex hallucinations are rarely occipital in origin. Instead, these generally arise from the temporal lobe or extracortical structures such as the brainstem and thalamus, although the posterior parietal lobe has also been implicated [7,8]. While insight retention can vary during episodes of hallucination, these false perceptions can negatively impact the patients' quality of life and place individuals in danger if not adequately managed [1].

## Case presentation

Appropriate protocols were followed, and consent was obtained by all participants in this study. This project was approved by the hospital's institutional review board. A 57-year-old man with a history of type 2 diabetes mellitus presented with sudden-onset, fully-formed visual hallucinations. He endorsed sudden-onset, isolated visual hallucinations that usually lasted a few minutes to almost an hour with each episode. He first noticed these apparitions while he was driving to work. Within the first few seconds of the symptom onset, he was confused by the false perceptions. The hallucinations served as a distraction and shifted his focus away from real objects on the road. Thus, his symptoms impacted his attention and posed a potential threat not only to himself but to other drivers as well. He described the appearance of multiple, normal-sized, and proportioned people standing or sitting in the highway median (left visual field). These people were colorful in nature but without otherwise distinct characteristics.

The hallucinations were non-threatening and silent. Although alarmed at their presence, he maintained an insight that the apparitions were not real. He estimated that this initial episode lasted between five and 20 minutes accompanied by a refractory period that lasted minutes to hours. While being examined in the hospital, his distorted visual perceptions had evolved and now incorporated other abnormal visual phenomena of a colorful “crystal’’ sheen on the walls, hallucinations of children on a playground outside the window of the second floor, facial metamorphopsia, left-sided proprioceptive distortions, and pixelation. Pixelation is the term used to describe blurry sections or fuzziness in an image due to the visibility of single-colored square display elements or individual pixels; this phenomenon is frequently encountered by patients experiencing various degrees of visual hallucinations [9,10]. He denied any associated complaints of headaches as well as prior psychiatric or neurological disease including seizures and symptomatic ischemic strokes. There was no history of childhood head trauma, former traumatic brain injury, or other obvious trigger factors. The patient had no family history of epileptic or psychiatric disorders. When taking into account collateral information obtained from the patient’s life-long partner, it was determined that the patient did not display epileptiform personality traits.

On examination, the patient was afebrile and hemodynamically stable; the neurological assessment was generally nonfocal, and there were no features indicating parkinsonism. The patient had normal cognition determined by a mini-mental state examination (MMSE). Although a mental status examination (MSE) was not performed, his insight was never compromised, and no obvious delusions or disorganized thought processes were noted. Thus, further neuropsychiatric evaluations were not warranted at that time. A thorough infectious and metabolic workup of the blood and cerebrospinal fluid (CSF) was performed (Tables 1, 2). Pertinent positives included initially elevated blood glucose of 465 mg/dL; however, symptoms neither responded to nor improved after glycemic control, nor with a dopaminergic blockade.

**Table 1:** Serum laboratory tests and results

Laboratory Test	Result
Complete blood count	Grossly normal
Complete metabolic panel	Grossly normal
Blood glucose	465 mg/dL
Blood culture	Negative for growth

**Table 2:** CSF laboratory tests and results RBC: Red blood cells; WBC: White blood cells; CSF: Cerebrospinal fluid.

Laboratory Test	Result	Reference Range
Opening pressure	11 cm H_2_O	6-20 cm H_2_O
Color	Clear	Clear/Colorless
Glucose	290 mg/dL	40-70 mg/dL
Protein	25 mg/dL	23-38 mg/dL
RBC	0	<5
WBC	<3	<5
Gram stain	Negative	Negative
Culture	No growth	No growth

**Figure 1:**
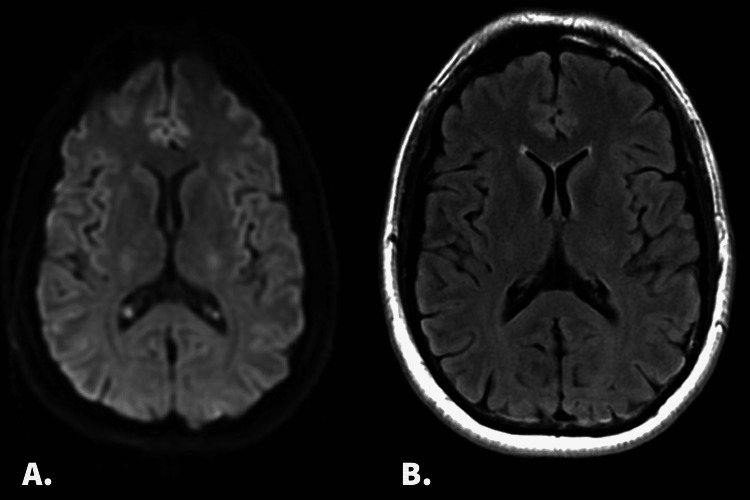
Patient’s MRI imaging sequences The diffusion-weighted imaging (DWI) sequence in Panel A is without diffusion restriction that would suggest an acute or subacute ischemic infarct. The T2 sequence in Panel B is without significant changes that would suggest prior ischemic infarcts or underlying chronic microvascular white matter disease.

A magnetic resonance imaging (MRI) of the brain was unrevealing for focal explanatory lesions (Figure 1). The patient underwent a total of 96 hours of continuous-electroencephalogram (CEEG) monitoring. When the recording was first started, he was in an awake, non-drowsy, ictal state as evidenced by a posterior dominant rhythm (PDR) without gross abnormalities. No photic stimulation or sleep deprivation was conducted.

**Figure 2:**
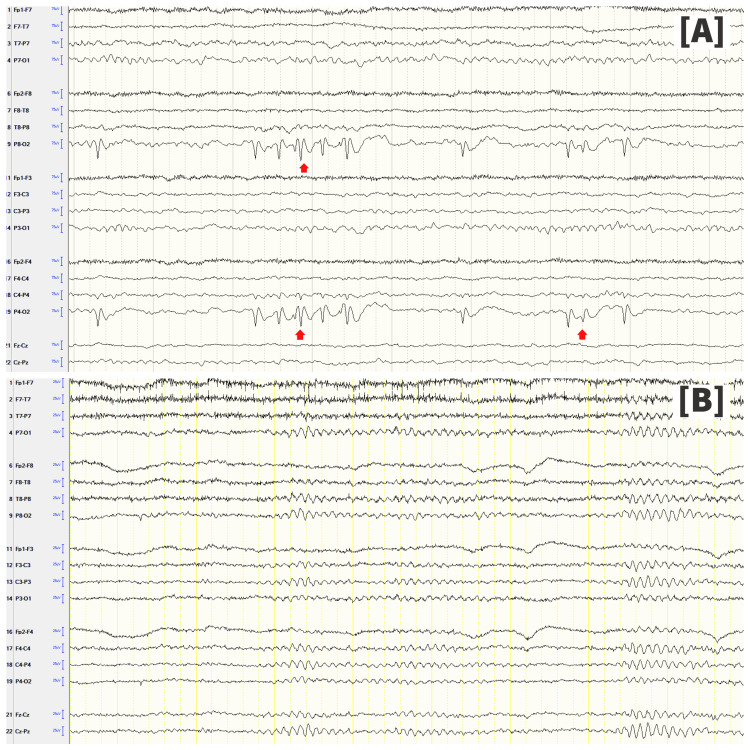
Patient’s double montage electroencephalograms Double montage EEG in Panel A show unilateral, right-sided occipital spikes in the absence of field spread to surrounding regions (arrows). Normalization of double montage EEG in Panel B is achieved after AED administration indicating successful treatment. EEG: Electroencephalogram; AED: Antiepileptic drug.

CEEG monitoring revealed frequent, focal right occipital lobe abnormal electrical activity correlating with the presentation of visual hallucinations (Figure 2, Panel A). He subsequently received a 60 mg/kg loading dose of levetiracetam and was initially started on 1,000 mg of levetiracetam twice daily as maintenance. The patient was noted to have prolongation in the time interval between hallucinations and effectively a decrease in the hallucination frequency. Complete resolution and full success in treatment, as evidenced by a grossly normal electroencephalogram (EEG) and no further altered visual perception, were achieved through up-titration of the antiepileptic drug (AED) to 1,500 mg twice daily and within 16 hours from initial AED administration (Figure 2, Panel B). Of note, there was no history of chronic kidney or liver disease that would warrant switching medications or render changes in dosage based on metabolism and excretion of the AED, nor did he have a known psychiatric history that would have us consider an alternative AED.

## Discussion

While visual hallucinations initiated in the occipital lobe are commonly elementary in nature, there have been reports of combined simple and complex visual hallucinations in the setting of occipital lobe epilepsy. This phenomenon is attributed to epileptic activity that initiates from the occipital lobe followed by spreading to the temporal lobe resulting in complex hallucinations [5,11]. When spreading occurs, simple occipital lobe hallucinations progress to display characteristic features of temporal lobe involvement. These include auditory features accompanied by lapses in memory and a heightened sense of fear [12].

Visual hallucinations and their etiology can also be categorized into irritative or release forms [13]. Traditionally, irritative forms are easily localized and present with simple hallucinations, and the individual lacks insight regarding false perceptions [12]. Often, the irritation is localized to the primary visual cortex (Brodmann's area 17), and pathologies associated with the irritative form include migraines, tumors, and epilepsy [13].

In contrast, the pathology associated with the release form is often thought to involve the visual association cortices (Brodmann's areas 18 and 19) and can result in more intricate hallucinations [14]. Under normal conditions, input to the visual association cortices is regulated by inhibitory processes. Thus, deafferentation in these cortices results in disinhibition and subsequent hyperactivity, giving rise to the release phenomenon [15]. Visual hallucinations of this form provide little to no localizing value and are described as detailed, complex scenes [16]. Patients are often aware of their false perceptions and, therefore, are in less distress when compared to the irritative form [12].

Lesions anywhere along the afferent visual pathway or the accessory pathway ascending from the brainstem can result in the release phenomenon. In the visually impaired, loss of visual input leads to the "release" of the visual cortex resulting in a form of phantom vision hallucinations known as Charles Bonnet syndrome (CBS) [15]. Similarly, loss of the accessory inhibitory influence arising from the midbrain, thalamus, and brainstem is thought to explain the syndrome of peduncular hallucinosis (PH) [16]. Patients with PH often describe vivid, complex, and colorful hallucinations characteristically involving animals and people that vary from one episode to the other [17]. Any region of the visual field may be involved [15]. Additional symptoms associated with damage and/or compression of nearby structures such as oculomotor palsy, hemiparesis, and gait ataxia can also be observed [18]. A comprehensive review found that the majority of PH results from ischemic infarct followed by mass lesions [18]. Insight is commonly retained in both CBS and PH [15].

Visual phenomena are often erroneously assumed to be occipital in origin without consideration for other possible origins [7]. In our case, EEG findings suggested focal occipital lobe epileptiform activity by evidence of unilateral, occipital spikes in the absence of field spread to surrounding regions, despite the patient experiencing a mix of both simple and complex hallucinations. Complex hallucinations as a result of focal occipital seizures differ from prior reports. In a study conducted by Bien et al., individuals exhibiting well-localized, occipital seizures described experiencing elementary hallucinations, illusions, or visual loss but never complex hallucinations. Furthermore, the individuals that reported experiencing complex hallucinations were found to have occipitotemporal or anteromedial temporal lobe onset [7].

Along with the lack of temporal lobe involvement on EEG, typical temporal lobe features failed to be demonstrated, making the spread of seizure activity a less likely explanation for this atypical presentation. While EEG suggests seizures, traditionally categorized as irritative, further characterization of the hallucinations suggests a more complex etiology with seemingly more release-like features [13]. No form of vision loss or impairment was reported in our patient, ruling out CBS. Given the hallucination complexity, along with retained awareness, we suggest possible underlying PH in addition to occipital epilepsy. Despite unrevealing MRI findings, injury to the brainstem or thalamus cannot be definitively ruled out in this case. A 1.5-Tesla MRI was used with plans for the patient to undergo repeat imaging via a 3-Tesla MRI as an outpatient, but the patient was unfortunately lost to further follow-up.

Furthermore, we suggest the theory of lesion-induced effects on network connections between structures, rather than the individual structures themselves. We propose that damage to connections between the occipital lobe and brainstem may manifest in overlapping symptoms of both occipital lobe hallucinations and PH. Case reports have described PH-like symptoms secondary to cortical parietal lesions in the absence of brainstem or thalamic lesions [19]. However, to our knowledge, this is the first reported case of PH-like symptoms secondary to cortical pathology specific to the occipital lobe. Network-based localization studies have shown that the shared symptoms, despite differing locations, are likely due to the overlap in their network connectivity [20]. Functional neuroimaging of these networks and their physiologic connectivity is difficult due to the inherent damage to the connections themselves resulting in symptoms. While complex hallucinations are not yet well studied, this possibly explains our patient with an unusual presentation of occipital lobe seizures with features of PH.

## Conclusions

Peduncular hallucinosis is a rare neurological phenomenon typically characterized by vivid, brightly colored visual hallucinations of people and objects. Most frequently, lesions are localized to the thalamus, midbrain, and brainstem. In this study, we showed this phenomenon occurring in a case of focal right occipital seizures suggesting a network-based localizing lesion. Furthermore, it emphasizes the importance of brain interconnections, rather than isolated brain regions when evaluating the possible etiology of visual hallucinations. Despite the lack of definitive confirmation, this case demonstrates important differentials to be considered in patients presenting with complex visual hallucinations in the absence of an auditory component.
